# Squamous Cell Carcinoma of the External Auditory Canal Presenting as a Persistent Ear Infection: A Case Report and Imaging Features

**DOI:** 10.7759/cureus.66188

**Published:** 2024-08-05

**Authors:** Jayanthraj Gone, Tyler Fontaine, Faraz Eshaghi, Mohammed Z Rehman

**Affiliations:** 1 Radiology, Hospital Corporation of America (HCA) Florida Bayonet Point Hospital, Hudson, USA; 2 Radiology, Hospital Corporation of America (HCA) Florida Trinity Hospital, Trinity, USA; 3 Internal Medicine, Hospital Corporation of America (HCA) Florida Bayonet Point Hospital, Hudson, USA

**Keywords:** facial nerve, temporomandibular joint, middle cranial fossa, middle ear, external auditory canal, squamous cell carcinoma

## Abstract

Squamous cell carcinoma (SCC) is the most common malignant tumor involving the temporal bone but generally very rare. The temporomandibular joint (TMJ), middle cranial fossa, and facial nerve canal are uncommon areas for the tumor to spread. We present the case of primary SCC of the temporal bone in a 63-year-old male presenting for otorrhea, otalgia, facial weakness, and facial pain after failing outpatient antibiotic therapy for an ear infection. Initial inpatient workup was significant for a hypertensive emergency, leukocytosis, and acute kidney injury. Opacification of cavities (i.e., left middle ear, external auditory canal (EAC)), destructive bony changes (i.e., mastoiditis, erosion of facial nerve canal, and TMJ), and invasion of the middle cranial fossa due to a soft tissue mass were noted on CT and MRI. Operative biopsy showed moderately differentiated SCC. The patient received treatment at the hospital consisting of antibiotics and supportive treatment. Plans for an outpatient PET scan and chemoradiotherapy per consultants' recommendations were arranged. The patient was discharged with appropriate medications and outpatient referrals and underwent infuse-a-port placement. Overall, this case describes some key points given the limited studies thus far. It demonstrates certain imaging characteristics of SCC of the temporal bone in the setting of a chronic ear infection. The malignancy spreads to the posterior TMJ wall and the temporal lobe, which very few cases have shown. The tumor also invades specifically the mastoid and tympanic segments of the facial nerve canal. This may be one of the first cases to showcase these features given the rarity of their simultaneous occurrence.

## Introduction

Malignant tumors of the temporal bone are extremely rare, accounting for less than 0.2% of all head and neck malignancies [1]. These tumors mostly affect patients of age 60 or older and have a male predilection (about 60% of cases) [2]. Squamous cell carcinoma (SCC) is the most common one involving the area, accounting for roughly 40% of cases [3]. SCC of the temporal bone is divided into two categories depending on its site of origin: primary (i.e., external auditory canal (EAC), middle ear, mastoid) or secondary (i.e., auricular skin, periauricular skin, parotid gland, skull base) [1,4]. Risk factors include radiation exposure, otitis media, otitis externa, cholesteatoma, human papillomavirus, and sun exposure [5]. Clinical features most commonly include otalgia, otorrhea, and hearing loss [6]. However, such symptoms are also observed in conditions like otitis media, otitis externa, and mastoiditis causing an overlap in presentation and mistaking cancer for an inflammatory disease [7,8]. Skull base osteomyelitis, both the typical and atypical forms, can present with similar nonspecific symptoms as well despite occurring commonly at an advanced stage in immunocompromised patients [7]. The lack of response to antimicrobial therapy and irrigation helps raise suspicion, but symptoms usually present for several months, making early diagnosis difficult [9]. Meanwhile, the tumor can rapidly grow without detection, possibly invading further structures like the parotid gland, facial nerve, brain, and neck [10,11]. CT usually shows a hypodense, poorly marginated mass possibly with destructive osseous changes and opacification of cavities [12]. MRI can further appreciate homogeneous enhancement, mass effect, and erosive soft tissue changes [13,14]. Otoscopic examination along with imaging features provides a preliminary diagnosis, while tissue biopsy confirms the diagnosis [6]. Management involves surgical resection, radiotherapy, chemotherapy (most commonly cisplatin, 5-fluorouracil, and docetaxel), immunotherapy, and auditory rehabilitation [15]. PET can assess tumor stage and prognosis is variable depending on factors like site of origin, tumor spread, and treatment response [2]. Though the American Joint Committee on Cancer (AJCC) staging system is used for most head and neck malignancies, the Pittsburgh staging system (PSS) is most commonly used for primary temporal bone malignancies [6]. Our case shows the invasion of SCC particularly to the mastoid and tympanic segments of the facial nerve canal, posterior temporomandibular joint (TMJ) wall, and temporal lobe. Not many cases have demonstrated this combination of features thus far.

## Case presentation

**Figure 1:**
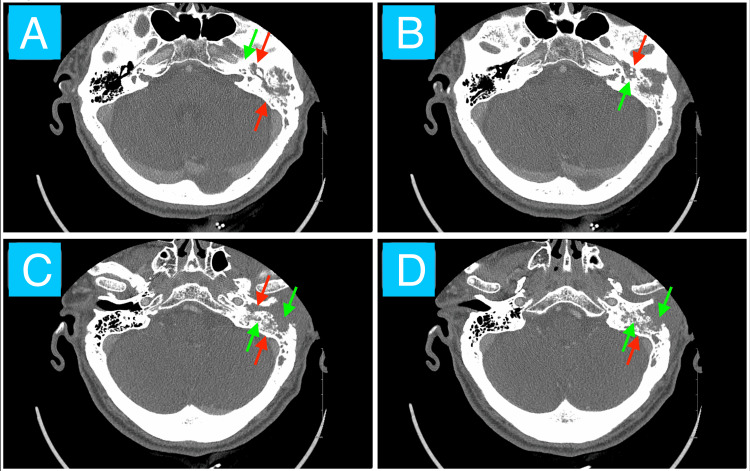
Non-contrast CT of the head Axial CT of the head without contrast from most superior (image A) to most inferior (image D) in a 63-year-old male showing cavitary opacification (left middle ear, EAC, mastoid bone) (red arrows) and destructive bony changes (coalescent mastoiditis, erosion of mastoid and tympanic segments of facial nerve canal) (green arrows). These findings are suspicious for an aggressive mass. Biopsy confirmed moderately differentiated SCC. EAC: external auditory canal; SCC: squamous cell carcinoma

A 63-year-old male with a past medical history of a tobacco use disorder, former alcohol use, ischemic cerebrovascular accident (CVA), paroxysmal atrial fibrillation, lumbar spinal stenosis, hypertension, and hyperlipidemia presented to the emergency department for left ear pain with yellow-green and bloody discharge, left facial pain, and left facial nerve palsy (House-Brackmann grade IV) for two months. He was diagnosed with a left ear infection and failed outpatient amoxicillin three months ago. The patient denied any known history of diabetes. Initial vitals and labs showed hypertensive emergency, leukocytosis, and acute kidney injury. The CT imaging without contrast showed opacification of the left middle ear, EAC, and mastoid air cells due to a soft tissue mass (Figure 1). These findings were associated with destructive changes to the temporal bone along with the mastoid and tympanic segments of the facial nerve canal (Figure 1).

**Figure 2:**
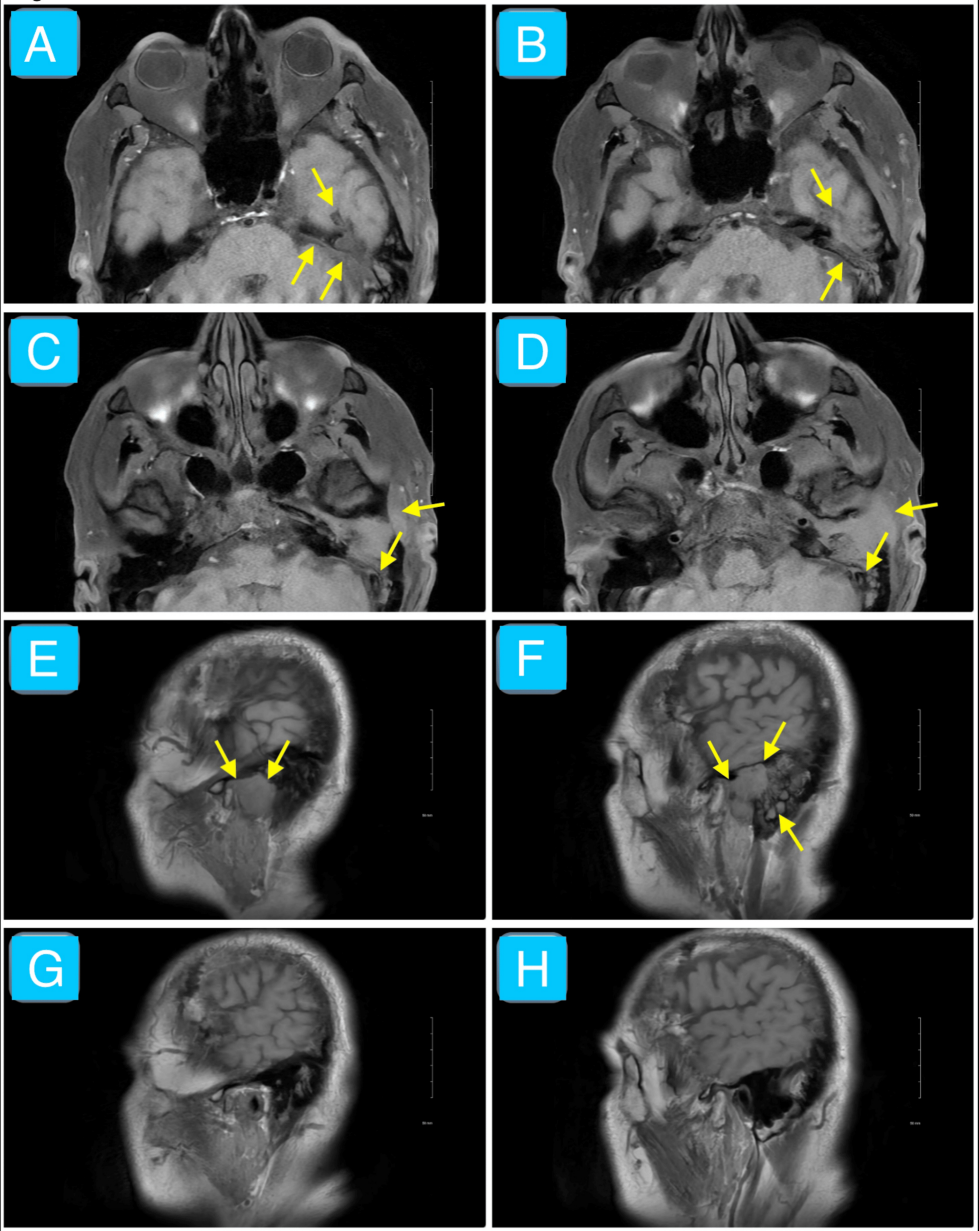
Non-contrast T1-weighted MRI of the face Axial (images A-D) and sagittal (images E-F (left ear), G-H (right ear)) T1-weighted sequence MRI of the face without contrast in a 63-year-old male showing a 3.6 × 1.8 × 3.0 cm poorly defined, mostly homogeneous, hypointense lesion invading the left EAC, middle ear, petrous apex, mastoid bone, posterior TMJ wall, temporal lobe, facial nerve canal, and retromandibular space (yellow arrows). These findings are consistent with an aggressive mass. Biopsy confirmed moderately differentiated SCC. EAC: external auditory canal; TMJ: temporomandibular joint; SCC: squamous cell carcinoma

The MRI with and without contrast showed a 3.6 × 1.8 × 3.0 cm mass mostly in the left EAC with contrast enhancement extending through the middle ear into the left petrous apex, mastoid air cells, TMJ, temporal lobe, and retromandibular space (Pittsburgh stage T4) (Figure 2). These findings were associated with EAC and posterior TMJ wall erosion and invasion of the left middle cranial fossa (Figure 2).

Ear bacterial cultures showed normal respiratory flora. The patient was admitted for concerns of chronic suppurative otitis media, malignant otitis externa, mastoiditis, and skull base osteomyelitis. Management consisted of intravenous (IV) antibiotics (vancomycin and piperacillin-tazobactam), ciprofloxacin ear drops, wound care, and appropriate home and supportive medications. Otolaryngology noted a mass completely occupying the auditory canal and invading the posterior and superior EAC surfaces via operative examination of the left ear. The mass was debrided, and multiple specimens of it were used for a frozen section exam, which revealed an invasive moderately differentiated SCC. Infectious disease transitioned antibiotics from IV to oral route given negative cultures despite poor long-term outcome. Oncology recommended an outpatient PET scan and plans for chemoradiotherapy (cisplatin, 5-fluorouracil, and docetaxel) given that the patient was not a candidate for surgical intervention. The patient was discharged with outpatient referrals for specialists given clinical improvement and underwent an infuse-a-port placement via ultrasound and fluoroscopy guidance without postprocedural complications. Tentative plans to monitor for recurrence and progression included follow-up CT imaging with contrast at three-, six-, 12-, and 24-month intervals.

## Discussion

**Figure 3:**
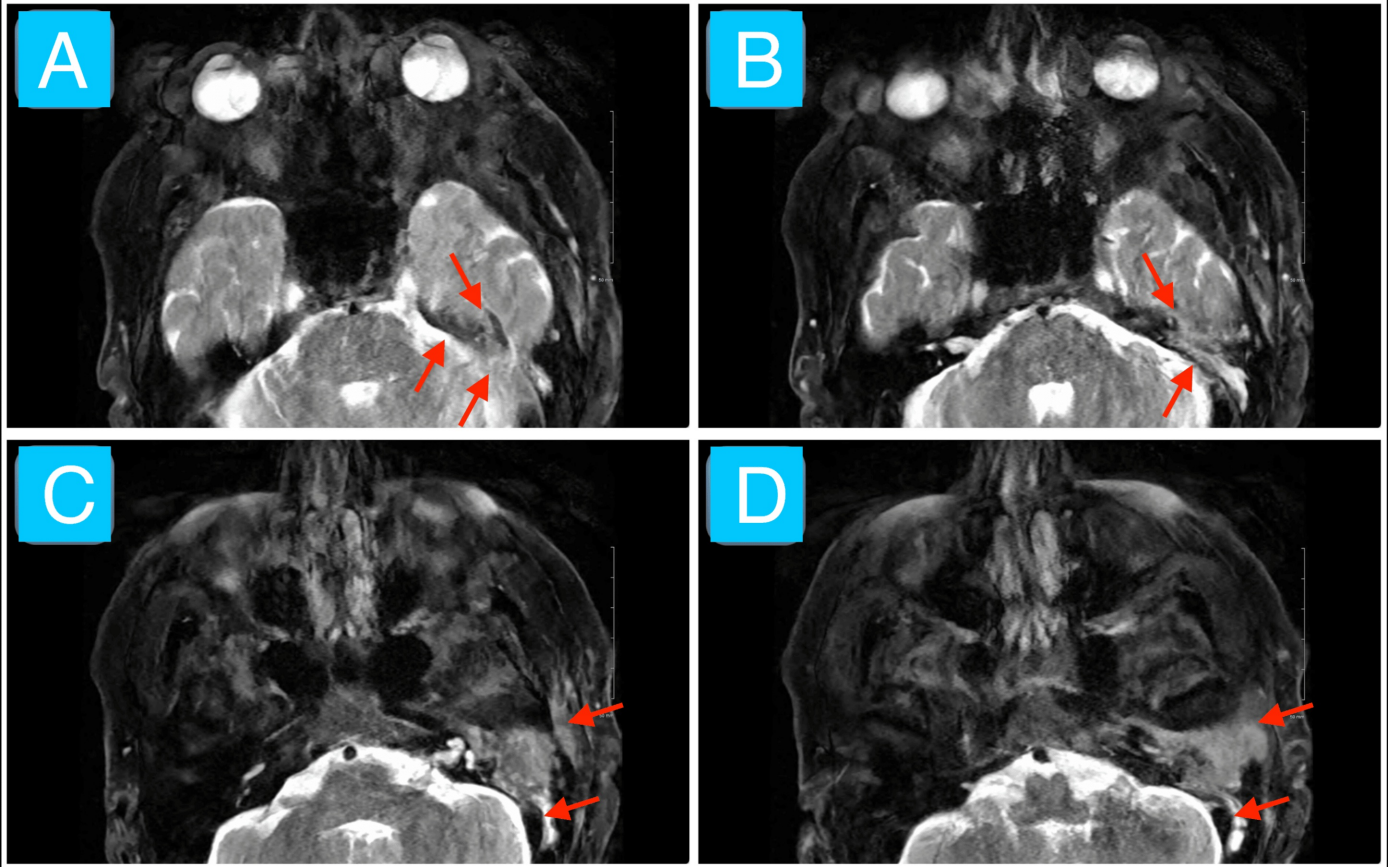
Non-contrast T2-weighted MRI of the face Axial T2-weighted sequence MRI of the face without contrast from most superior (image A) to most inferior (image D) in a 63-year-old male showing a 3.6 × 1.8 × 3.0 cm poorly defined, mostly homogeneous, hyperintense lesion invading the left EAC, middle ear, petrous apex, mastoid bone, posterior TMJ wall, temporal lobe, facial nerve canal, and retromandibular space (red arrows). These findings are consistent with an aggressive mass despite motion artifact. Biopsy confirmed moderately differentiated SCC. EAC: external auditory canal; TMJ: temporomandibular joint; SCC: squamous cell carcinoma

**Figure 4:**
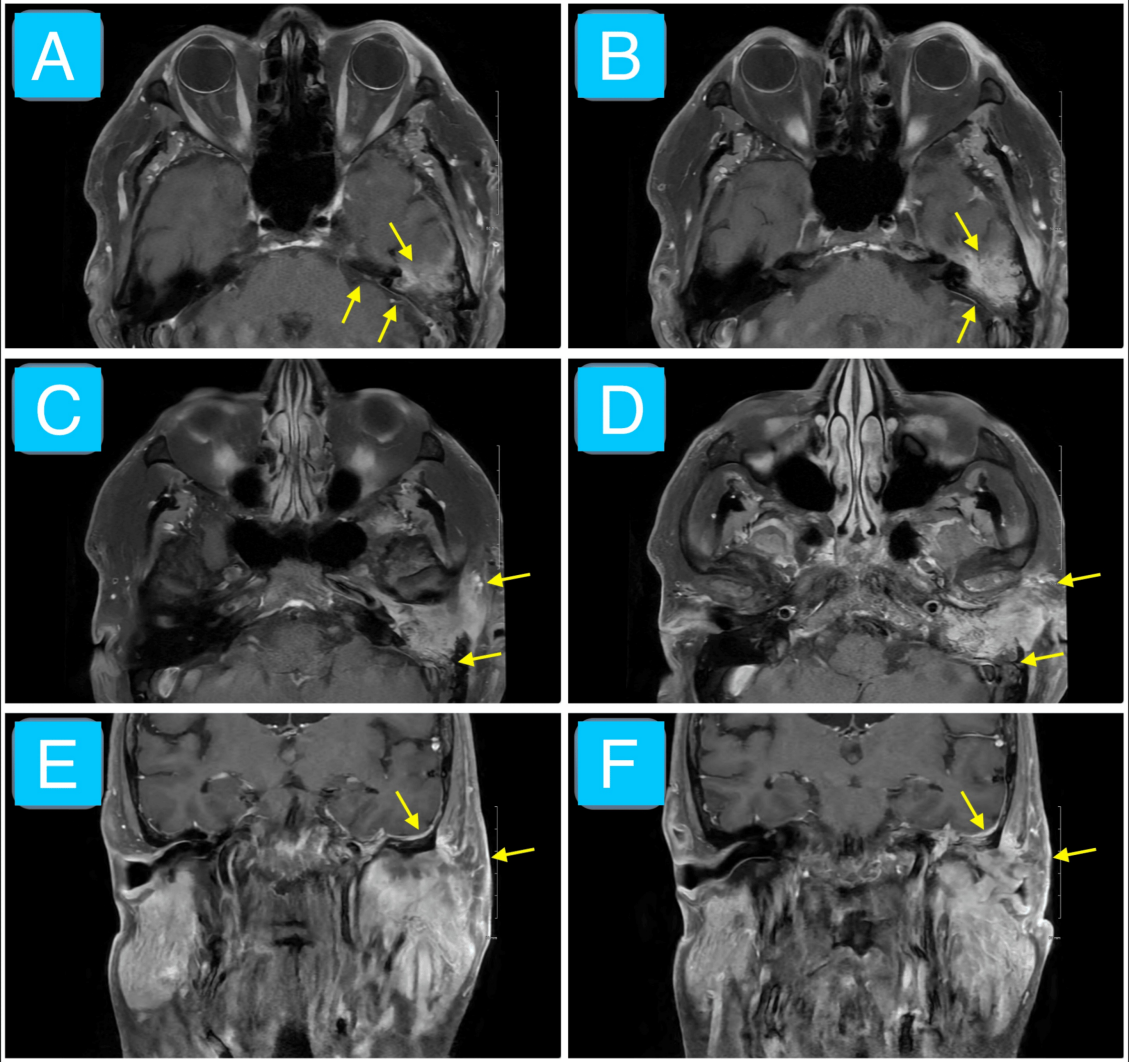
Contrast-enhanced T1-weighted MRI of the face Post-contrast axial (images A-D) and coronal (images E-F) T1-weighted sequence MRI of the face in a 63-year-old male showing a 3.6 × 1.8 × 3.0 cm continuously enhancing lesion invading the left EAC, middle ear, petrous apex, mastoid bone, posterior TMJ wall, temporal lobe (with local dural enhancement), facial nerve canal, and retromandibular space (yellow arrows). These findings are consistent with an aggressive mass. Biopsy confirmed moderately differentiated SCC. EAC: external auditory canal; TMJ: temporomandibular joint; SCC: squamous cell carcinoma

We have presented a case of SCC of the left temporal bone region in the setting of a chronic persistent left ear infection (chronic suppurative otitis media and malignant otitis externa). Initial evaluation revealed purulent and sanguineous otorrhea, otalgia, left facial nerve palsy, left facial tenderness to palpation, and auricular erythema and edema. These findings corroborate the difficulty in distinguishing infectious and neoplastic otologic diseases [7,8]. The CT imaging without contrast overall shows evidence of opacification of the left middle ear, EAC, and mastoid air cells (Figure 1). Specific bony changes noted include coalescent mastoiditis and erosion of tympanic and mastoid segments of the facial nerve canal (Figure 1). The main differential diagnoses at this juncture included a phlegmon (given that symptoms occurred chronically and no distinct borders of the mass exist in the setting of an infection) and excessive granulation tissue (likely a keloid given its potential to grow over weeks to months in response to tissue injury) [12,13]. The MRI shows a mostly homogeneous mass with poorly defined borders mainly occupying the left EAC despite extending into the adjacent structures mentioned in the case presentation, including the left TMJ, temporal lobe, and two facial nerve canal segments (Figures 2-4). Most cases of SCC of the temporal bone, despite limited studies, are relatively hypo- and hyperintense on T1- and T2-weighted MRI without contrast, respectively, which is echoed by this case (Figures 2-3) [14]. The T1-weighted MRI with gadolinium contrast shows diffuse enhancement of the mass and dural enhancement close to it (Figure 4). A phlegmon or keloid would most likely show heterogeneous enhancement, which indicates that the mass is less likely such and more likely an aggressive tumor [12,13]. Additional differential diagnoses included SCC, basal cell carcinoma, cutaneous angiosarcoma, and ceruminous adenocarcinoma given the similar gross appearance and invasive nature of such tumors [4]. Biopsy confirmed moderately invasive SCC. The mass likely originated from the cutaneous part of the EAC given that the patient described his initial symptom as an itch in that region and the imaging demonstrates that it mostly occupies the EAC (Figures 2-4). No universally accepted radiological signs seem to have been established yet for SCC of the EAC [13,14]. 

Overall, this case highlights some important points. An uncommon feature of SCC of the EAC is TMJ involvement (4% of cases as reported by one study), which is shown in this case [10]. The tumor abuts against (and slightly invades) the temporal lobe and partially obliterates the facial nerve canal (mastoid and tympanic segments), which is very rare. Despite cases of each separate phenomenon, only a few cases captured all three together. Acharya et al. presented a case with a similar history and clinical presentation except the presence of a mastoid fistula due to an ulcer and the lack of facial nerve palsy (which was present in our case) [3]. Ouaz et al. also presented a unique case of SCC with a painless mastoid skin ulceration [4]. None of the aforementioned cases have shown SCC of the EAC specifically invading towards the temporal lobe. Limitations of our specific case report include the lack of audiological examinations and the motion artifact on MRI, which could have better characterized the clinical scenario and imaging features of the patient, respectively. Additional studies are necessary to learn more about the consistency of metastatic patterns of the malignancy. One important recommendation for clinicians is that one ought to look out for all adjacent areas where SCC can metastasize despite the rarity of each specific invasive pattern.

## Conclusions

Malignant neoplasms involving the temporal bone, especially SCC of the EAC, represent a miniscule percentage of head and neck tumors. They may be hard to distinguish from infectious, wound repair, and other malignant disorders of the ear given the similar clinical presentations. The occurrence of tumor extension to the middle cranial fossa, TMJ, and facial nerve canal simultaneously is extremely rare. This case portrays such an instance in a middle-aged male in the setting of a persistent ear infection, which is an interesting learning point. The malignancy reaches the temporal lobe with the adjacent dura mater, posterior TMJ wall, and two facial nerve canal segments. A tissue biopsy established the diagnosis of moderately differentiated SCC. The general characteristics of SCC appearance on imaging can be seen in this case despite the lack of established radiological signs and the necessity for additional studies (i.e., to emphasize common potential sites for metastasis). This could be the first case to describe the SCC of the EAC spreading to the aforementioned locations, which most likely indicates that the patient has a poor prognosis. Additional studies are needed to find out more about metastatic patterns of the malignancy and the variety of clinical feature combinations. Malignant tumors, though very uncommon, should be considered as differential diagnoses early to initiate appropriate management promptly.
